# BBS4 and BBS5 show functional redundancy in the BBSome to regulate the degradative sorting of ciliary sensory receptors

**DOI:** 10.1038/srep11855

**Published:** 2015-07-07

**Authors:** Qingwen Xu, Yuxia Zhang, Qing Wei, Yan Huang, Yan Li, Kun Ling, Jinghua Hu

**Affiliations:** 1 Department of Nephrology and Hypertension, Mayo Clinic, Rochester, Minnesota, USA; 2 Mayo Translational PKD Center; 3 Department of Biochemistry and Molecular Biology, Mayo Clinic, Rochester, Minnesota, USA

## Abstract

Cilia harbor sensory receptors for various signaling cascades critical for vertebrate development. However, the mechanisms underlying the ciliary homeostasis of sensory receptors remain elusive. Here, we demonstrate that BBS-4 and BBS-5, two distinct BBSome components, show unexpected functional redundancy in the context of cilia in *C. elegans*. BBS-4 directly interacts with BBS-5 and the interaction can be disrupted by a conserved mutation identified in human BBS4. Surprisingly, we found that BBS-4 and BBS-5 act redundantly in the BBSome to regulate the ciliary removal, rather than the ciliary entry or retrograde IFT transport, of various sensory receptors. Further analyses indicate that co-depletion of BBS-4 and BBS-5 disrupts the lysosome-targeted degradative sorting of ciliary sensory receptors. Moreover, mammalian BBS4 and BBS5 also interact directly and coordinate the ciliary removal of polycystin 2. Hence, we reveal a novel and highly conserved role for the BBSome in fine-tuning ciliary signaling by regulating the ciliary removal of sensory receptors for lysosomal degradation.

Sensory transduction capabilities of cilia are highly conserved across species. Cells utilize primary cilia to receive environmental stimuli that are converted into physiological responses. Primary cilia harbor sensory receptors for various signaling cascades that are critical for normal organ development and tissue pattern formation, such as polycystin, Sonic hedgehog (shh), Wnt, and PDGF pathways^1^,^2^. With rapid advancements during the past decade in the positional cloning of human disease genes, a wide variety of genetic disorders, such as polycystic kidney disease (PKD), Bardet-Biedl syndrome (BBS), Joubert syndrome (JBTS), nephronophthisis (NPHP), and Meckel-Gruber syndrome (MKS), have been characterized molecularly as cilia-related diseases, or ciliopathies^3^,^4^,^5^. Consistent with the presence of cilia on most cell surfaces, most ciliopathies occur as syndromic disorders that affect many organs during development, including the kidneys, central nervous system (CNS), eyes, liver, limbs, cardiovascular system and fat storage tissue. Despite the physiological and clinical relevance of cilia, the molecular mechanisms that regulate cilia function and biogenesis as well as the connections between disease gene functions and pathology remain largely elusive.

As the sensory organelle for most eukaryotic cells, the cilium requires a stringent sorting mechanism to selectively regulate the entry and removal of various ciliogenic proteins. Clearly, ciliary membrane receptors must be precisely located and regulated to endow cilia with their specific sensory properties and must be fine-tuned both temporally and spatially to execute their cellular functions. The mechanisms regulating ciliary membrane localization likely involve targeting, import, retention, and endocytic/degradative removal. Improper ciliary receptor localization could cause sensory transduction defects and human ciliopathies. We previously proposed that a downregulation process involving ubiquitination and lysosome-targeted degradation regulates the ciliary localization and signaling of polycystins in *C. elegans*^6^,^7^. Whether the downregulation acts in a common manner for other ciliary receptors as well as its correlation with ciliopathies remain unclear yet.

BBS is an autosomal recessive ciliopathy with 19 causal loci being reported to independently cause the disorder^8^,^9^. Eight evolutionarily conserved BBS genes (BBS1, 2, 4, 5, 7, 8, 9 and 18) encode proteins that form a complex termed the BBSome^9^,^10^,^11^. The BBSome shares the similar structural elements with clathrin coats, COPI, and COPII, and has been proposed to polymerize and recognize the ciliary membrane proteins^12^,^13^. However, experimental data from different model organisms/systems suggest distinct, sometimes controversial, roles for the BBSome in regulating the localization of membrane or membrane-associated proteins. For example, in *C. reinhardtii*, loss of the BBSome causes abnormal accumulation of several membrane-associated signaling proteins due to the defective IFT-dependent BBSome-mediated export from the cilium^14^,^15^. Knockdown of *bbs* genes in zebrafish leads to progressive cilia loss in ciliated organ Kupffer’s vesicle defective retrograde melanosome transport^16^. In mammalian systems, dysfunctional BBSome compromises the ciliary import of several sensory receptors^12^,^17^,^18^. Interestingly, abnormal ciliary accumulation of sensory receptors was also observed in either *Bbs* KO mice or BBSome-disrupted cells^19^,^20^,^21^,^22^,^23^,^24^. The abnormal accumulation of ciliogenic proteins in BBSome-deficient cilia is believed to be caused by compromised retrograde IFT transport, which is in good agreement with the observations made in *C. elegans* that the BBSome regulates the integrity and/or assembly of Intraflagellar Transport (IFT) particles^25^,^26^,^27^. However, to date, the definite molecular activity of the BBSome in regulating the homoeostasis of ciliary membrane proteins remain unclear.

Our previous findings indicated that the BBSome acts as an important player regulating IFT assembly and its turnaround in cilia^25^. However, neither *bbs-4* nor *bbs-5* single mutant show cilia-related defects in *C. elegans*. Here, we demonstrated that BBS-4 and BBS-5, two distinct BBSome components without shared domains, unexpectedly play redundant role in the context of cilia. *bbs-4; bbs-5* double mutant show compromised IFT integrity as observed in other *bbs* mutants. BBS-4 and BBS-5 directly interact and the association is disrupted by a conserved mutation identified in human BBS4 patients. Interestingly, all sensory receptors examined in our studies, including either IFT cargo OSM-9 or non-IFT cargo polycystin-2 and ODR-10, abnormally accumulate in *bbs-4; bbs-5* cilia, indicative of a non-IFT dependent role for the BBSome in regulating the proper localization of ciliary receptors. Similar defects were also observed in *bbs-7* mutants. We further demonstrated that the abnormal accumulation of ciliary sensory receptors in *bbs* mutants is due to the compromised lysosome-targeted degradative sorting. Finally, we show that human BBS4 and BBS5 interact directly and function redundantly in downregulating ciliary polycystin-2. Thus, our data uncover an unexpected functional coordination between *BBS4* and *BBS5* in the context of cilia and reveal a highly conserved role for the BBSome in downregulating sensory receptors from cilia for lysosome degradation.

## Results and Discussion

### BBS-4 and BBS-5 function redundantly in regulating ciliogenesis

**Figure 1:**
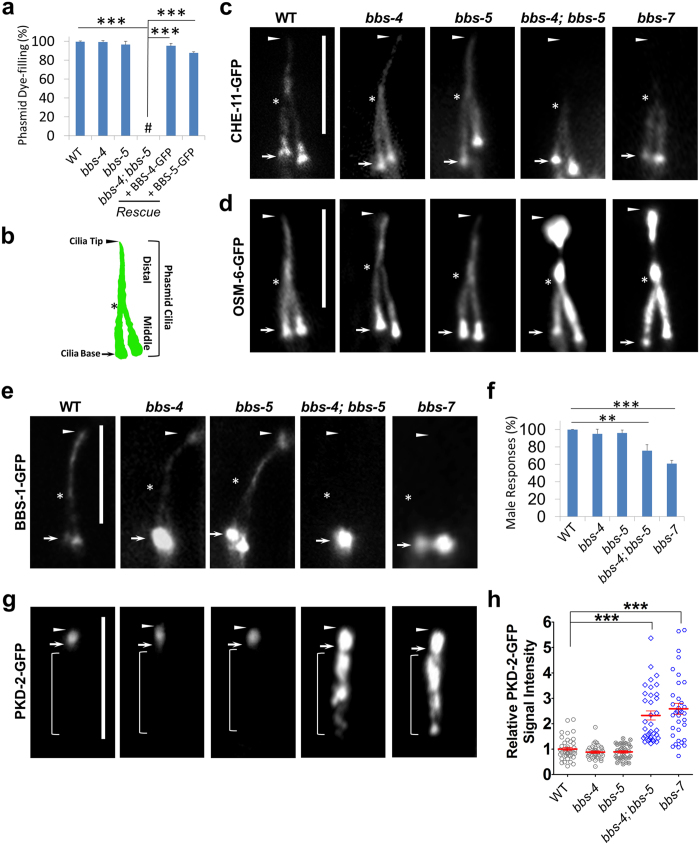
BBS-4 and BBS-5 play redundant roles in the context of cilia. **(a)** Dye-filling assay was used to examine ciliogenesis in worms. *bbs-4 (tm3038)* mutant and *bbs-5 (gk507)* mutant show normal ciliogenesis as the wild type (WT) worms. However, *bbs-4 (tm3038); bbs-5 (gk507)* double mutants are completely dye-filling minus, which could be fully rescued by introducing a wide copy of BBS-4-GFP or BBS-5-GFP. **#** denotes complete dye-filling minus. Results were represented as mean ± SD. **(b)** Schematic diagram of the phasmid cilia in worms. The phasmid has two sensory cilia whose tips bundle together. The axoneme of phasmid cilium contains the middle doublet and the distal singlet segment. In *bbs-4; bbs-5* double and *bbs-7* single mutants, **(c)** GFP-tagged IFT-A component CHE-11 is absent in distal segments, **(d)** GFP-tagged IFT-B component OSM-6 show strong accumulation at cilia tip, and **(e)** BBS-1 is absent from whole cilia. **(f)** *bbs-4; bbs-5* mutants show defective mating behavior. **(g)** PKD-2 mislocalizes and accumulates in *bbs-4; bbs-5* mutant cilia. Results were represented as mean ± SD. **(h)** Relative fluorescence intensities for PKD-2-GFP signal in sensory cilia were dot plotted. PKD-2 level increases more than 2 fold in *bbs-4; bbs-5* cilia when compared to that in WT animals. Results represented as mean ± SEM. Arrows and arrowheads indicate the base and tip of cilia, respectively. Stars note the junction of middle and distal segment. Brackets indicate the dendrite of polycystin-expressing sensory neuron. Data represent three or more experiments. In each experiment, n > 40 were used in each group. ***p < 0.001; **p < 0.01. Scale bars, 5 μm.

Our previous findings identified the BBSome as an important player in regulating the assembly of IFT machinery in *C. elegans*^25^. To study the *in vivo* roles of BBS proteins, we rigorously tested all available null alleles of worm *bbs* genes. Dye-filling assay is routinely used to examine the biogenesis of worm cilia^28^. Interestingly, unlike other *bbs* mutants that show defective ciliogenesis, *bbs-4* or *bbs-5* single mutants are completely normal in dye-filling assay, indicating that BBS-4 or BBS-5 alone is dispensable for ciliogenesis (Fig. 1a). To further test if BBS-4 and BBS-5 are functionally redundant, we generated *bbs-4; bbs-5* double mutants. Remarkably, we found that *bbs-4; bbs-5* double mutants show typical cilia defect as observed in other *bbs* mutants (Fig. 1a). Moreover, introducing a wild-type copy of *bbs-4* or *bbs-5* gene into *bbs-4; bbs-5* could fully restore cilia biogenesis (Fig. 1a). BBS4 is a multiple tetratricopeptide repeats (TPR) containing protein, whereas BBS5 is a pleckstrin homology (PH) domain-containing protein (Fig. S1a and ref.12). It is thus unexpected that two BBSome components that share no similar protein domains (Fig. S1a and S1b) can function redundantly in the context of cilia.

We then asked whether the defects observed in *bbs-4; bbs-5* mutants are BBSome-dependent or not. In *C. elegans*, the canonical heterotrimeric kinesin-II and homodimeric OSM-3 (the ortholog of human KIF17) coordinate anterograde IFT transport in cilia^26^,^29^. *C. elegans* phasmid cilia contain two distinct segments, middle doublet and distal singlet segments (Fig. 1b). Slower Kinesin-II and faster OSM-3 move the same IFT particle along the middle doublet at intermediate speed 0.7 μm/s, and then OSM-3 kinesin alone moves the IFT particle along the distal singlet at faster speed 1.3 μm/s^26,29^. Dysfunctional BBSome results in the dissociation between IFT-A and IFT-B, which leads to that IFT-B-OSM-3 subcomplex moves at 1.3 μm/s in anterograde IFT along the whole axoneme, whereas IFT-A-Kinesin-II subcomplex is restricted only in middle doublets and moves at 0.5 μm/s in anterograde IFT^26^,^29^. Due to the essential role of IFT-A as retrograde IFT machinery, the absence of IFT-A in distal singlets causes the accumulation of IFT-B components in *bbs* mutants^25^,^26^. By examining IFT-A component CHE-11 (the ortholog of human IFT140) and IFT-B component OSM-6 (the ortholog of human IFT52), we found that *bbs-4; bbs-5* and *bbs-7* share similar mutant phenotypes in that CHE-11 is absent, but OSM-6 abnormally accumulates, in the distal segments of plasmid cilia (Fig. 1c,d). Furthermore, IFT analyses confirmed that, similar to *bbs-7*, *bbs-4; bbs-5* cilia exhibit abnormal anterograde IFT velocities in that IFT-A moves at slower speed (~0.5 μm/s) and IFT-B moves at faster speed (~1.3 μm/s) along the middle doublet (Fig. S2). When the BBSome is disrupted, individual BBS proteins also fail to enter cilia^25^,^30^. As expected, we observed that GFP-tagged BBS-1 lost ciliary entry in *bbs-4; bbs-5* mutants (Fig. 1e). Taken together, these observations indicate that the reported role of the BBSome in regulating IFT integrity is impaired in *bbs-4; bbs-5* double mutants.

### BBS-4 and BBS-5 act redundantly in regulating the cilia removal of various ciliary sensory receptors

We then asked whether BBS-4 and BBS-5 also act redundantly in cilia signaling in *C. elegans*. Polycystins, which are mutated in Autosomal dominant polycystic kidney disease (ADPKD), are conserved mechanosensory receptors on cilia across ciliated species^31^. In *C. elegans*, mating behavior assays are used as simple readouts to test polycystin-mediated cilia signaling^32^. Similar to what we observed in ciliogenesis assay, *bbs-4; bbs-5* double mutants, but not *bbs-4* or *bbs-5* single mutants, exhibit defective polycystin signaling similar to that observed in *bbs-7* mutants (Fig. 1f), suggesting a coordinated role for BBS-4 and BBS-5 in regulating cilia signaling. Mammalian BBSome is well known to regulate the ciliary import of ciliary sensory receptors^12^,^17^,^18^. We then asked whether BBS-4 and BBS-5 regulate the ciliary entry of PKD-2 (the worm ortholog of human polycystin-2). GFP-tagged PKD-2 localizes to the cilia of male-specific RnB sensory neurons to mediate mechanosensory function in *C. elegans*^32^. In WT animals, PKD-2 majorly localizes inside RnB cilia in male tails (Fig. 1g). Surprisingly, contratry to our prediction that PKD-2 fails to enter cilia, we observed almost 3 fold increasing of PKD-2 signal inside cilia (Fig. 1g,h). Abnormal PKD-2 accumulation was also found along dendrite fragments adjacent to cilia base (Fig. 1g). Similar PKD-2 accumulation was reported in male head CEM neurons in *bbs-7* mutants^33^.

**Figure 2:**
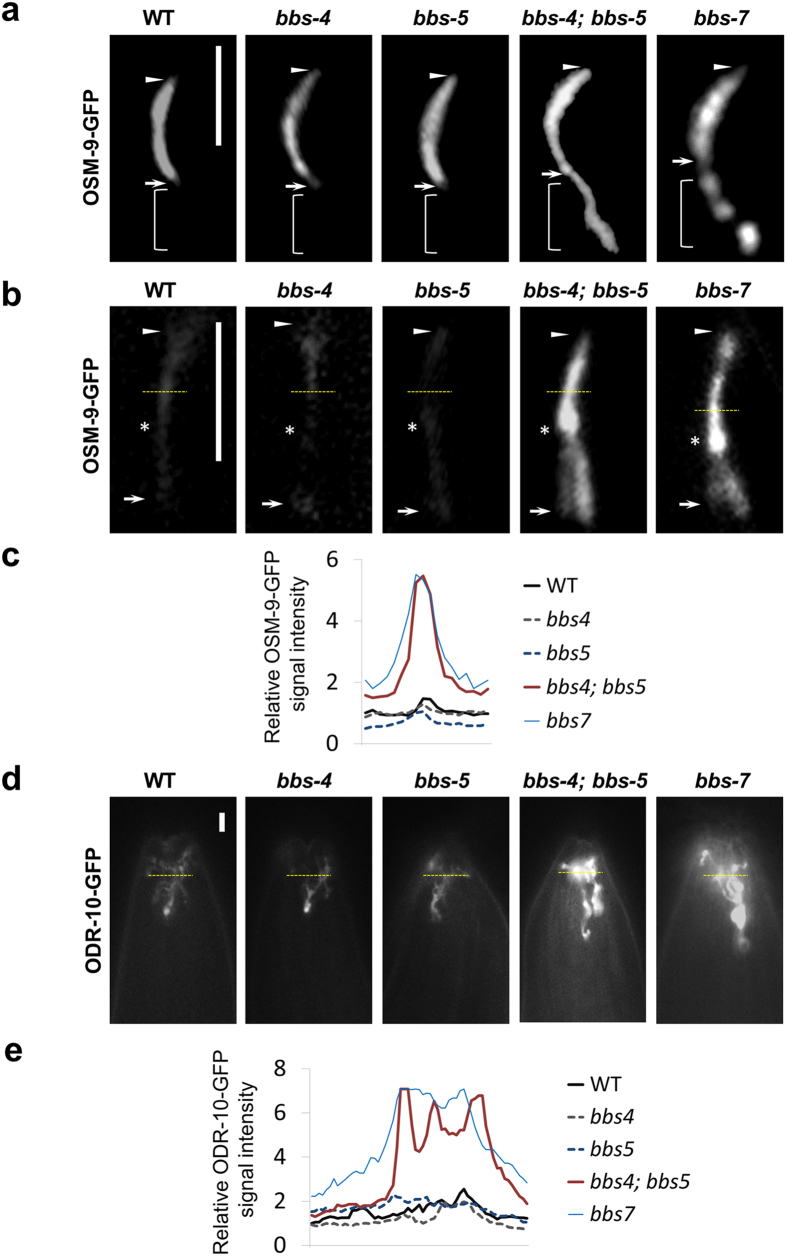
Mislocalization and accumulation of OSM-9 and ODR-10 in *bbs-4; bbs-5* and *bbs-7* mutant cilia. **(a)** OSM-9, the TRP mechanosensory receptor channel, accumulates both inside and below OLQ cilia in *bbs-4; bbs-5* and *bbs-7* mutants**. (b)** OSM-9 accumulates inside phasmid cilia in *bbs-4; bbs-5* and *bbs-7* mutants. **(c)** Relative fluorescence intensity of OSM-9-GFP in each group shown in (b) was plotted. Yellow dashed line indicates the pixels used to measure the fluorescence intensity. **(d)** GPCR ODR-10 accumulates inside AWA cilia in *bbs-4; bbs-5* and *bbs-7* mutants. **(e)** Relative fluorescence intensity of ODR-10-GFP in each group shown indicated as a yellow dashed line in **(d)** was plotted. Arrows and arrowheads indicate the base and tip of cilia, respectively. Stars note the junction of middle and distal segment. Scale bars, 5 μm.

To further determine whether the abnormal accumulation in *bbs-4; bbs-5* cilia is a PKD-2-specific defect or a general defect for other sensory receptors, we examined two additional sensory receptors, the mechanosensory receptor TRP channel OSM-9^34^ and the G protein-coupled receptor ODR-10^35^. GFP-tagged OSM-9 strongly labels the cilia of mechanosensory OLQ neurons and phasmid neurons, and GFP-tagged ODR-10 labels the fan-shaped cilia of chemosensory AWA neurons (Fig. 2). Remarkably, both OSM-9 and ODR-10 show strong accumulations, with ~6-fold increasing of protein levels in their native expressing cilia in *bbs-4; bbs-5* mutants (Fig. 2). We thus concluded that BBS-4 and BBS-5 act redundantly to coordinate the proper cilia homeostasis of various sensory receptors. Moreover, the observation that comparable accumulation of PKD-2, OSM-9, and ODR-10 in *bbs-7* cilia suggests the involvement of the whole BBSome in this process (Fig. 1h and 2).

Given the discoveries that the BBSome promotes the ciliary entry of several sensory receptors in cultured mammalian cells^12^,^13^,^17^,^18^, it is thus an unexpected observation that, in *bbs-4; bbs-5* worms, all sensory receptors examined, including PKD-2 (Fig. 1g), OSM-9 (Fig. 2a,b), and ODR-10 (Fig. 2d), still enter and even accumulate in cilia. We reasoned that the ciliary accumulation of sensory receptors could be caused by either increased cilia entry or disrupted cilia removal. To rule out the possibility that BBSome-deficient cilia may have defective or leaky diffusion barriers at cilia base that would lead to uncontrolled ciliary entry for membrane proteins, we examined several other native membrane proteins at cilia base, including PPK-1 (the homolog of human phosphatidylinositol-4-phosphate 5’ kinase), Y57G11C.37 (the homolog of human membrane protein anoctamin 8), and phosphodiesterase PDE-1. None of them show abnormal cilia entry or accumulation in *bbs-4; bbs-5* mutants (Fig. S3 and S4a), suggesting that the diffusion barrier at cilia base is not disrupted in BBSome-deficient cilia. The transition zone (TZ) protein MKS-5 also shows normal localization in *bbs-4; bbs-5* mutants, suggesting that the TZ forms properly at cilia base (Fig. S4b). We thus concluded that the accumulation of sensory receptors in BBSome-deficient cilia is probably caused by defective cilia removal.

### BBS-4 directly interacts with BBS-5 and the association is impaired by a conserved mutation identified in *BBS4* patient

**Figure 3:**
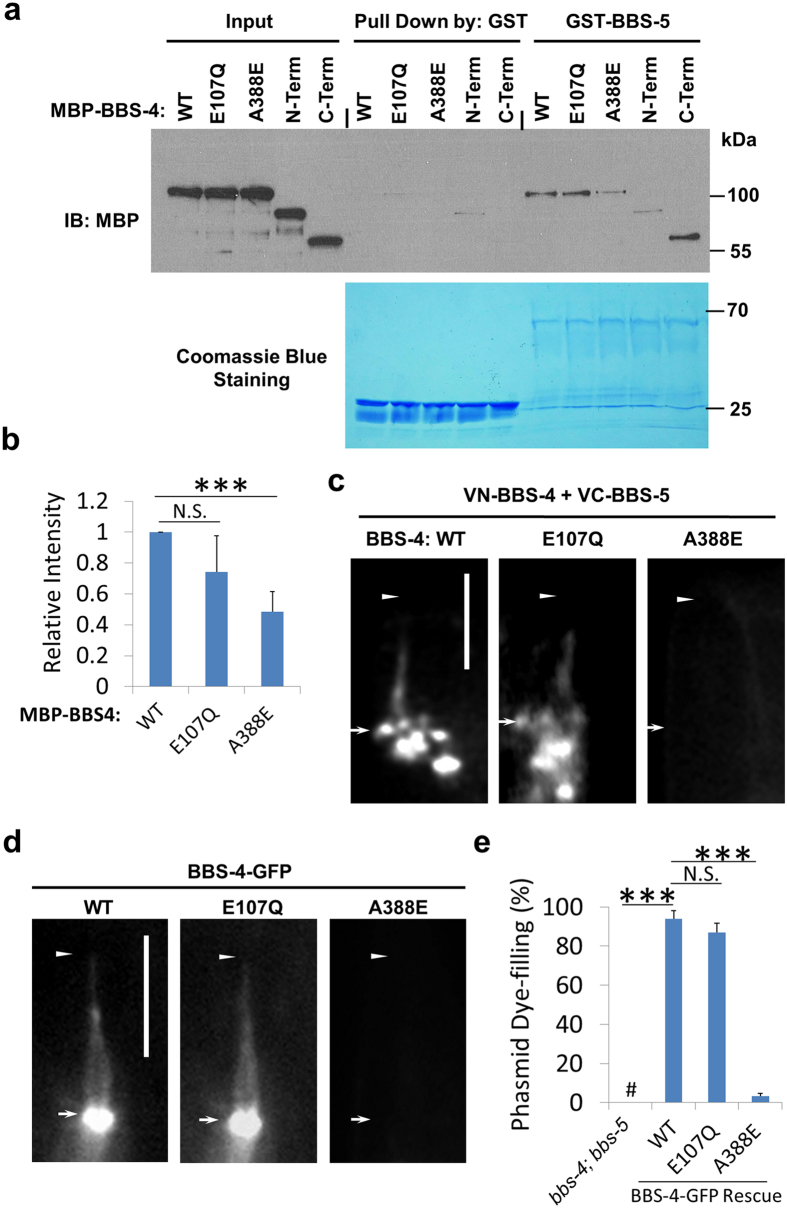
BBS-4 and BBS-5 directly associate, and the interaction is abolished by a conserved ciliopathy mutation identified in BBS4 patients. (**a**) BBS-4 directly interacts with BBS-5 via its carboxyl-terminus. GST pull down assay was performed to determine the association between GST fused BBS-5 and MBP fused BBS-4 variants. BBS-4 wild type (WT), BBS-4 with two human ciliopathy related mutations (E107Q and A388E) and BBS-4 fragments (N-Term, amino-terminus, a.a. 1-280; C-Term, carboxyl-terminus, a.a. 270-462) were included. Upper panel, blotted with anti-MBP antibody. Lower panel, loading of GST and GST-BBS5 proteins was shown by Commassie Blue staining. (**b**) BBS-4-BBS-5 interaction was impaired by ciliopathy mutation A388E in BBS-4. The results shown in (a) were quantified. (**c**) BiFC was used to visualize the *in vivo* BBS-4-BBS-5 association. Stable fluorescence complementation between BBS-4 and BBS-5 was impaired by A388E, but not E107Q, mutation in BBS-4. (**d**) A388E, but not E107Q, compromises the ciliary targeting of BBS-4. Arrows and arrowheads indicate the base and tip of cilia, respectively. (**e**) BBS-4^A388E^ was unable to rescue ciliogenesis defect in *bbs-4; bbs-5* mutants. # denotes under-detectable dye-filling. Data represent three or more experiments. In each experiment, n > 40 were counted in each group. Results represented as mean ± SD. N.S., not statistically significant. ***p < 0.001. Scale bars, 5 μm.

It is believed that during the assembly of the BBSome, BBS2, 7, and 9 form the core, then BBS1, 5, 8, and finally BBS4 are added in a stepwise manner^13^,^36^. Given that BBS-4 and BBS-5 act redundantly and both are probably peripheral components of the BBSome, we hypothesized that BBS-4 and BBS-5 may directly interact with each other. Indeed, we observed the interaction in GST pull-down assay. We further mapped down the binding region in BBS-4 to the C-terminal 193 amino acids (a.a. 270–462) (Fig. 3a). To test whether BBS-4-BBS-5 association exists *in vivo*, we performed bimolecular fluorescence complementation (BiFC) assay in worms. BiFC assay was developed for direct visualization of protein-protein interaction in the same macromolecular complex in their natural environment, and we have successfully applied this approach to examine the *in vivo* association between IFT components within worm cilia^25^,^37^. As expected, strong fluorescence complementation between BBS-4 and BBS-5 was observed (Fig. 3c), indicative of *in vivo* BBS-4-BBS-5 association.

When examining the mutations reported in BBS4 patients, we found that 10 out of 11 missense mutation alleles in BBS4 patients locate to the C-terminal half of BBS4 protein, suggesting the importance of BBS4 C-terminus^38^. Intriguingly, we observed that the mutation at A388 in BBS-4 C-terminus (BBS-4^A388E^, mimicking the human pathogenic BBS4^A364E^ mutant^39^), but not the mutation at E107 in BBS-4 N-terminus (BBS-4^E107Q^, mimicking the human pathogenic BBS4^E85Q^ mutant^40^), significantly impaired the BBS-4-BBS-5 association in either *in vitro* GST pull-down (Fig. 3a,b) or *in vivo* BiFC (Fig. 3c) assays. In BiFC assays, non-specific fluorescence complementation can be occasionally observed in cell bodies (likely in the ER or the Golgi during translation) for BiFC-tagged protein pairs. Similar fluorescence signal in the cell body was also observed for BBS4^A364E^-BBS-5 pair, suggesting that both BiFC-tagged proteins were expressed successfully but could not form stable complex in cilia (Fig. S5a). Further studies on GFP-tagged BBS-4 mutant proteins showed that BBS-4^A388E^, but not BBS-4^E107Q^, lost ciliary targeting (Fig. 3d and S5b). Additionally, BBS-4^E107Q^, but not BBS-4^A388E^, can rescue the ciliogenesis defect in *bbs-4; bbs-5* mutants (Fig. 3e). Consistent with our observations in worms that the N-terminal E107Q mutation shows little impact on BBS-4 function as well as its association with BBS-5, the pathogenicity of human BBS4^E85Q^ mutant is rather weak, which only causes a mild retinal-specific phenotype resembling Leber’s congenital amaurosis (LCA) but lacks more severe defects in the kidneys and other vital organs that associated with typical *BBS4* (including *BBS4*^*A388E*^) patients^40^. Whether the impact on BBS4-BBS5 association is a critical factor that determining the pathogenicity of mutations identified in *BBS4* patients will be interesting to be explored.

### BBS-4 and BBS-5 downregulate ubiquitinated sensory receptors from cilia

Loss of the BBSome or ARL6/BBS3 causes abnormal accumulation of signaling proteins in *Chlamydomonas flagella*^14^,^15^ and several sensory receptors in mammalian cilia^19^,^20^,^21^,^22^,^23^,^24^,^25^. It is proposed that the compromised retrograde IFT transport in BBSome-deficient cilia can cause the accumulation of IFT cargoes inside mammalian cilia^14^,^15^,^19^,^20^,^25^. However, for the sensory receptors used in current study, PKD-2 and ODR-10 do not show any IFT movement inside cilia and have been reported as non-IFT cargoes^41^. Also, OMS-9 shows no detectable IFT movement in OLQ cilia and only weak IFT movement in phasmid cilia (data not shown). These facts challenge the existing working model and suggest that the ciliary accumulation of sensory receptors in BBSome-deficient cilia is likely a non-IFT-dependent defect. Notably, we previously reported that a lysosome-targeted downregulation process is used to adjust PKD-2 ciliary homeostasis in *C. elegans*^6^,^7^. The mislocalization pattern of PKD-2 in *bbs-4; bbs-5* mutants (Fig. 1g) actually recapitulates that in *stam-1* mutants, in which the depletion of early endosome component STAM-1 prevents ubiquitinated PKD-2 from being targeted for degradative sorting^6^. Taken these into consideration, we hypothesized that BBS-4 and BBS-5 may play a redundant role in the BBSome to regulate the degradative sorting of ciliary sensory receptors.

**Figure 4:**
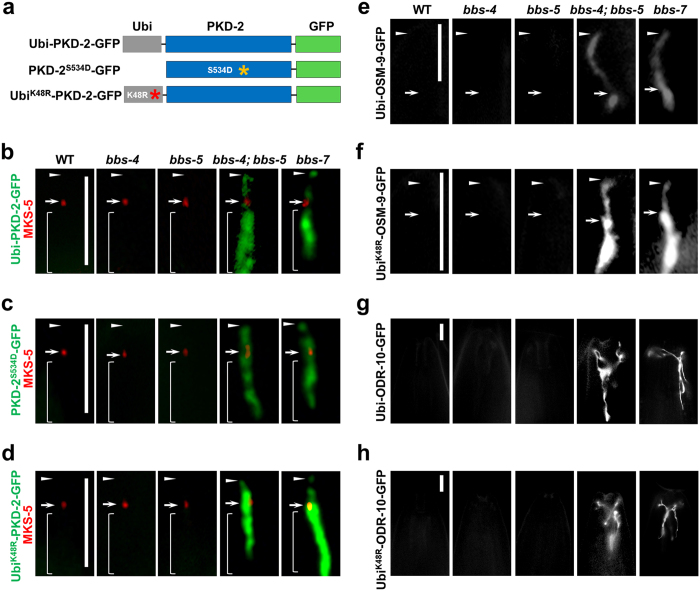
BBS-4 and BBS-5 function redundantly in the downregulation of sensory receptors from cilia. **(a)** Cartoons illustrating the constructs expressing Ubi-PKD-2, Ubi-PKD-2^S534D^ or Ubi^K48R^-PKD-2 proteins. All constructs were tagged with GFP to visualize in live animals. **(b-d)** The degradative sorting of Ubi-PKD-2, Ubi-PKD-2^S534D^, and Ubi^K48R^-PKD-2 are compromised in *bbs-4; bbs-5* double and *bbs-7* single mutants. mCherry-tagged MKS-5 was co-expressed as a transition zone marker to label the ciliary base. **(e-f)** The degradative sorting of Ubi-OSM-9 and Ubi^K48R^-OSM-9 are compromised in amphid OLQ cilia in *bbs-4; bbs-5* double and *bbs-7* single mutants. **(g-h)** The degradative sorting of Ubi-ODR-10 and Ubi^K48R^-ODR-10 are compromised in amphid AWA fan-shaped cilia in *bbs-4; bbs-5* double and *bbs-7* single mutants. Arrows and arrowheads indicate the cilia base and tip, respectively. Brackets indicate distal dendrites. Scale bars, 5 μm.

Direct conjugation of an ubiquitin to the target protein has been successfully used as a tool to study the endocytic removal of membrane proteins^6^,^42^. We previously reported that Ubi-PKD-2-GFP (a GFP tagged PKD-2 with an ubiquitin conjugated to the amino terminus, see Fig. 4a) was largely absent from cilia due to the enhanced degradative sorting^6^. As expected, Ubi-PKD-2-GFP was not detected in WT, *bbs-4* or *bbs-5* cilia (Fig. 4b). However, in *bbs-4; bbs-5* or *bbs-7* mutants, Ubi-PKD-2-GFP strongly accumulated both inside and below cilia (Fig. 4b), suggesting that the degradative sorting of ubiquitinated PKD-2 is compromised in these *bbs* mutants. The detectable signal for Ubi-PKD-2-GFP inside cilia indicates that the ciliary import of receptors is not disrupted in *bbs-4; bbs-5* or *bbs-7* mutants. We reported that Casein Kinase 2 modulates PKD-2 activity by phosphorylating its S534 site^7^. PKD-2^S534D^ mutant protein mimics the constitutively phosphorylated PKD-2 at S534 site and may represent a hyperactive PKD-2 channel^7^. Similar to ubiquitinated PKD-2, PKD-2^S534D^ is absent from *WT* cilia due to the enhanced degradative sorting^6^,^7^. In good agreement with the observations made with Ubi-PKD-2-GFP, the downregulation of PKD-2^S534D^-GFP from cilia was also disrupted in *bbs-4; bbs-5* or *bbs-7* mutants (Fig. 4c).

Next, we asked whether the BBSome regulates the lysosome-targeted or proteasome-targeted degradation. Mono-ubiquitinated proteins are targeted for lysosomal degradation, whereas proteasomal degradation requires the formation of poly-ubiquitin chains at ubiquitin K48 site^43^. We found that Ubi^K48R^-PKD-2-GFP, which harbors a K48R mutation in ubiquitin (Fig. 4a) and is incapable to be poly-ubiquitinated^43^, shares similar mislocalization pattern with Ubi-PKD-2-GFP in all strains examined (Fig. 4d), suggesting that the BBSome acts upstream of lysosomal degradation of PKD-2. We further fused ubiquitin or ubiquitin^K48R^ to OSM-9 and ODR-10. Similar to Ubi-PKD-2, all ubiqutien conjugated receptors strongly accumulate in *bbs-4; bbs-5* or *bbs-7* mutants, but are absent in *WT*, *bbs-4* or *bbs-5* single mutants (Fig. 4e–h). We thus concluded that BBS-4 and BBS-5 play redundant role in the BBSome to ubiquitously regulate the lysosome-targeted degradation of ciliary sensory receptors.

**Figure 5:**
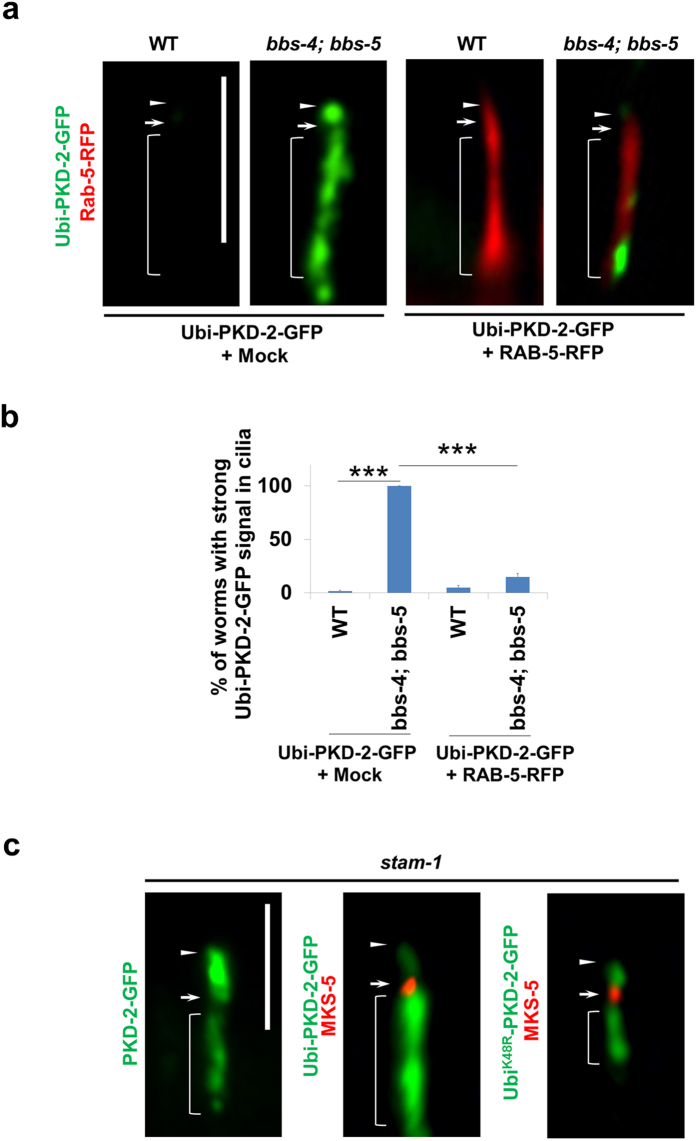
BBS-4 and BBS-5 downregulate PKD-2 through lysosomal degradation pathway. **(a)** Enhancing lysosomal degradation by overexpressing RAB-5 significantly reduces PKD-2 accumulation in *bbs-4; bbs-5* cilia. **(b)** The results shown in **(a)** were quantified. Data represent three or more experiments. **(c)** Disruption of lysosomal degradation in *stam-1* mutants leads to strong accumulation of WT PKD-2, Ubi-PKD-2-GFP, and Ubi^K48R^-PKD-2-GFP comparable to that observed in *bbs-4; bbs-5* double mutants (see Fig. 4). Arrows and arrowheads indicate the cilia base and tip, respectively. Brackets indicate distal dendrites. In each experiment, n > 40 were counted in each group. Results represented as mean ± SEM. ***p < 0.001. Scale bars, 5 μm.

Overexpression of early endosome protein Rab5 can enhance endocytic traffic to lysosomes where polyubiquinated protein is efficiently degraded^44^. We reasoned that if the BBSome acts upstream of lysosomal degradation, overexpression of RAB-5 may restore the degradation defect in *bbs* mutants. As expected, we observed significant reduced ciliary accumulation of Ubi-PKD-2-GFP signal in *bbs-4; bbs-5* mutants upon the overexpression of RAB-5-RFP, indicative of restored degradative sorting (Fig. 5a,b). In contrary, depletion of early endosomal protein STAM-1 leads to strong accumulation of PKD-2-GFP, Ubi-PKD-2-GFP, and Ubi^K48R^-PKD-2-GFP (Fig. 5c). Taken together, these observations suggest that the BBSome acts upstream of the early endosome, probably in endocytic stage at cilia base, to regulate the lysosomal sorting of ciliary receptors.

### The functional conservation for BBS4 and BBS5 in mammalian cells

**Figure 6:**
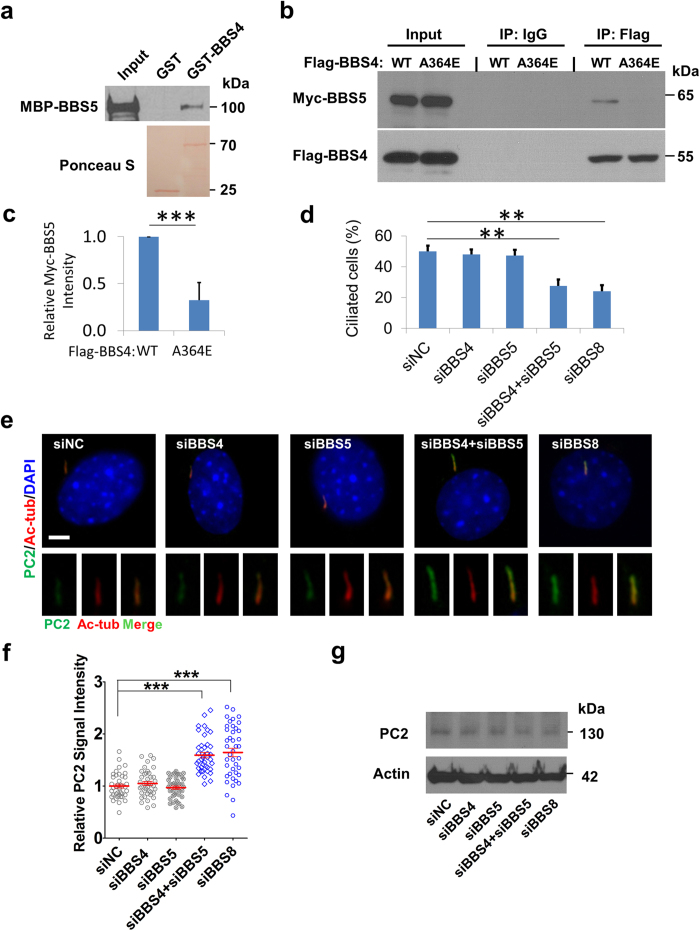
The redundant role of BBS4 and BBS5 in the context of cilia is highly conserved. **(a)** Human BBS4 protein directly interacts with BBS5. GST pull down assay between MBP fused human BBS4 and GST fused human BBS5. Upper panel, blotted with anti-MBP antibody. Lower panel, loading of GST and GST-BBS5 proteins was shown by Ponceau S staining. **(b, c)** The human ciliopathy mutation A364E in BBS4 significantly reduces the association between BBS4 and BBS5. HEK293T cells were co-transfected with Myc-tagged BBS5 and Flag-tagged BBS4 wild type (WT) or A364E mutant. The cell lysate was immunoprecipitated with normal mouse I gG or anti-Flag antibody followed by immunoblotting with anti-Flag and anti-Myc tag antibodies, respectively. Data are representative of three or more experiments. The relative amount of Myc-BBS5 or Myc-BBS5^A364E^ was quantified. Results represented as mean ± SD. ***p < 0.001. **(d)** BBS4 and BBS5 co-depletion impairs ciliogenesis in hTERT-RPE-1 (RPE) cells. The percentage of ciliated cells was shown as mean ± SD. Data represent three or more experiments. **p < 0.01. n>200. **(e, f)** BBS4 and BBS5 co-depletion cause ~2 fold increasing for the ciliary level of PC2 in ciliated RPE cells. Anti-Acetylated tubulin (Ac-tub) antibody was used to label cilia. DNA was stained with DAPI. Scale bar, 5 μm. Dot plot shows the relative fluorescence intensity of ciliary PC2. Bars in red indicate mean ± SEM. ***p < 0.001. n > 40. **(g)** PC2 protein level remains unchanged after knockdown of BBS4, BBS5 or BBS8 along, or knockdown of both BBS4 and BBS5. Cell lysates were analyzed for immunoblotting using indicated antibodies. Actin was used as a loading control.

Since all BBSome components are highly conserved across ciliated species during evolution, we asked whether our unexpected discoveries for BBS-4 and BBS-5 in *C. elegans* are also relevant in mammalian cells. We first validated the direct interaction between human BBS4 and BBS5 via GST pull-down assay (Fig. 6a). We then confirmed that Myc-tagged BBS5 could co-immunoprecipitate with Flag-tagged BBS4 after co-transfected into HEK293T cells (Fig. 6b). Similar to what we observed for worm proteins, introducing A364E mutation in BBS4 significantly reduced the association between BBS4 and BBS5 (Fig. 6b,c).

Next, siRNAs were designed to specifically target human *BBS4* and *BBS5* genes, and their knockdown efficiencies were validated by immunoblotting (Fig. S6a-d). hTERT-RPE-1 (human Retinal Pigmentosa Epithelial) cells treated with indicated siRNAs were serum-starved to induce ciliogenesis, followed by immunostaining with the cilia maker acetylated tubulin. As shown in Fig. 6d, in RPE cells, knockdown of *BBS4* or *BBS5* alone did not affect ciliogenesis, while depletion of both *BBS4* and *BBS5* significantly reduced ciliation ratio. Similar inhibition effect on ciliogenesis was observed by *BBS8* single knockdown (Fig. 6d). These results suggest that BBS4 and BBS5 also play redundant role to support ciliogenesis in mammalian cells.

To test whether BBS4 and BBS5 function redundantly in downregulating sensory receptors from mammalian cilia, we examined the localization of endogenous polycystin-2 (PC2) in RPE cells. Consistent with our observation in *C. elegans* (Fig. 1g,h), the ciliary intensity of PC2 significantly increased (~60%) in BBS4/BBS5 co-depleted or BBS8 depleted cells, but not in BBS4 or BBS5 depleted cells (Fig. 6e,f). Total protein level for PC2 was not altered in RNAi-treated cells (Fig. 6g).

**Figure 7:**
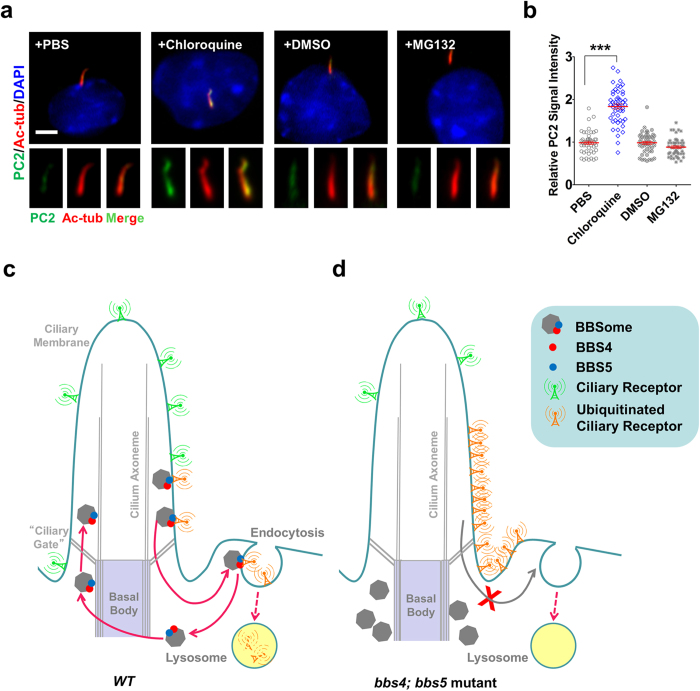
The BBSome regulates the removal of ciliary receptors for lysosomal degradation but not proteasomal degradation. **(a, b)** Treating with lysosomal inhibitor Chloroquine, but not proteasome inhibitor MG132, caused ~2 fold increasing for the ciliary level of PC2 in RPE cells. Anti-Acetylated tubulin (Ac-tub) antibody was used to label cilia. DNA was stained with DAPI. Scale bar, 5 μm. Data represent three or more experiments. Bars in red indicate mean ± SEM. ***p < 0.001. n > 40. **(c, d)** Working model for the BBSome in regulating the degradative sorting of ciliary sensory receptors.

Intriguingly, mammalian BBS4 has been implicated in regulating the proteasomal degradation of signaling mediators at cilia base^45^. To examine whether the ciliary accumulation of PC2 in BBSome-deficient RPE cells is caused by defective lysosomal degradation or proteasomal degradation, we treated the cells with the lysosomal inhibitor chloroquine or the proteasomal inhibitor MG132 at physiological relevant concentrations. Treatment of RPE cells with chloroquine, but not MG132, significantly increased cilia surface localization of PC2 protein by 2-fold (Fig. 7a,7b). Total protein level for PC2 was not altered after treating with chloroquine or MG132 (data not shown). We thus concluded that disrupted lysosomal degradation but not proteasomal degradation leads to the ciliary accumulation of PC2 similar to that observed in BBSome-deficient cells. In summary, these results suggest a redundant and conserved role for BBS4 and BBS5 in the BBSome in downregulating cilia receptors for lysosomal degradation (Fig. 7c,d).

## Discussion

Despite the physiological and clinical relevance of cilia, the molecular mechanisms that regulate cilia signaling, biogenesis, and the pathogenesis of ciliopathies remain poorly understood. Many studies have focused on the role of the BBSome in the context of cilia in regulating the ciliary entry of sensory receptors. Here, our results demonstrate the unexpected functional redundancy for two distinct BBSome components, BBS4 and BBS5, and reveal a novel role for the BBSome in the degradative sorting/removal of ciliary sensory receptors. We further confirmed that these phenomena are highly conserved across ciliated species. Cilia, as the antennae of the cell, transduce a plethora of sensory stimuli and must be fine-tuned both temporally and spatially^1^,^46^,^47^. Our results identify the BBSome as a key player in fine-tuning cilia signaling and advance our understanding about the pathogenesis of BBS disorders.

### How does the BBSome promote the degradative sorting of sensory receptors?

Ubiquitin-mediated receptor downregulation is used by cells for fine-tuning many signaling pathways, including growth factor receptors, GPCRs, immune receptors, and cell junction proteins^48^. To our surprise, the BBSome acts as a key determinant in receptor downregulation since none of BBSome components contains any known ubiquitin-binding motif. Further work will be required to determine whether and how the BBSome and/or its interactors recognize ubiquitinated cargo proteins. Another possibility is that the BBSome might not directly recognize ubiquitinated cargo, but rather sort downregulated receptors into an intermediate membrane compartment, then downregulated receptors will be ubiquitinated and recognized by degradative sorting machinery enriched around cilia base, such as STAM/Hrs^6^, Rab5^6^, Clathrin and AP2^49^. Notably, one recent study suggested that BBS4 genetically interacts with the endosomal sorting complexes required for transport (ESCRT) gene TSG101 and regulates the endosomal trafficking of Notch receptors^50^. It would be interesting to see whether ESCRT complex also acts in the degradative sorting of cilia sensory receptors. The enrichment of BBS proteins the ciliary compartment^25^ recently defined as endocytic-machinery-containing periciliary membrane compartment (PCMC)^49^ support this assumption. It is conceivable that this proposed intermediate membrane compartment could also function as a sorting site for the receptors that will be recycled for cilia entry. This functional model could explain why the BBSome is required for both the ciliary entry and removal of ciliogenic proteins.

### Implications for the pathogenesis Bardet-Biedl Syndrome

Our discoveries support the assertion that one major activity for the BBSome in the context of cilia is to precisely regulate the homeostasis of ciliary receptors by promoting the removal of activated or excess sensory receptors for lysosomal degradation. Dysfunctional BBSome leads to abnormal retention of sensory receptors inside cilia and could result in unwanted activation of ciliary signaling. The execution of cilia sensory capacities requires that signaling cascades of adequate strength and duration are generated from sensory receptors to elicit right responses. Just like the well-known case that excessive EGFR signaling poses a serious oncogenic threat^51^, excess cilia signaling could also be detrimental to tissue patterning and organogenesis. For example, in BBSome-deficient cells, accumulation of polycystins in kidney cilia could lead to aberrant calcium influx and uncontrolled cyst formation; and retention of Shh receptors in embryo cilia could lead to various developmental manifestations. In this regard, the BBSome may be a central player in negative feedback to restrain various ciliary signaling in time and space, which is key to ensuring that sensory outputs are adequate to the needs of the ciliated cell.

### Treating BBS as lysosomal disorders?

The disease mechanisms are still, to a large extent, unclear for various ciliopathies, which impedes the development of effective therapies. However, the implication of lysosomal degradation in the pathology of BBS has been hinted by emerging evidences from our and other labs, suggesting the potential of new therapeutic opportunities. The observation that overexpression of RAB-5 could promote the lysosomal degradation of accumulated polycystin-2 in BBSome-deficient cilia suggest that positive modulation of the downstream lysosomal system could be a novel therapeutic avenue for BBS disorders, and potentially other ciliopathies that share similar pathogenesis mechanism with BBS. Therapeutic interventions aiming at restoring lysosomal function, such as increasing the clearance capacity of lysosomes by small molecules, have yielded promising therapeutic effects for treating mouse models of various lysosomal disorders^52^,^53^. Similar strategy could be used to clear excess sensory receptors in BBSome-deficient cilia to restore the aberrant cilia signaling.

## Methods

### *C. elegans* mutant alleles and strains

Nematodes were raised using standard conditions. N2 worms represented wild-type animals in all assays described in this study. To ensure the expression levels of GFP markers comparable between the wild-type and mutant strains, we introduced each GFP transgene from the wild-type worms into individual mutant worms by genetic crossing. Dye-filling assay or polymerase chain reaction (PCR) was used to monitor the mutant genotype. All strains used in this study are listed in Table S1.

### Dye-filling assay

Worms were washed off the culturing plate with M9 buffer (3 g/L KH2PO4, 6 g/L Na_2_HPO_4_, 5 g/L NaCl and 1 mM MgSO_4_), collected by centrifugation at 500 g for 1 min, washed once with M9 buffer and then incubated in diluted DiI dye (D-282, Molecular Probes; 1:200 dilution in M9 of the 2 mg/mL stock solution in dimethyl formamide) for 1 h at room temperature. After incubation, the worms were washed at least three times with M9, transferred to an NGM plate without a bacterial lawn, and then observed under a fluorescence microscope (M2Bio, Zeiss).

### Mating behavior analysis

Multiple observation trials of male mating behavior were performed blindly using experimental and control animals in each trial. L4 transgenic males were isolated and maintained overnight at 15 °C. Mating behavior assays involved placing 1–4 virgin adult males on a newly seeded plate containing 10–12 *unc-31(e169)* adult hermaphrodites. The mating behavior of individual males was observed and response behavior was scored using the following criterion: a male who successfully responded to hermaphrodite contact within 5 min was scored as response positive (a male who failed to respond was negative). Responsiveness reflects the percentage of response positive males divided by the total number of males scored. Pair-wise comparisons were made using Mann–Whitney nonparametric and two-sided t-tests.

### Microscopy and imaging

Animals were raised at 20 °C. The live adult worms were mounted on 5% agar pads by anaesthetization in 10 mM levamisole (diluted in M9 buffer), observed, and images were acquired under an imaging microscope (Nikon TE 2000-U) with a Plan Apochromat 60 X (NA 1.49) oil immersion objective (Nikon).

### IFT measurement

All IFT analyses were performed in phasmids for easier observation. IFT motility was observed using Apochromat 100 × (NA 1.49) oil immersion total internal reflection fluorescence objective (Nikon). Motility stacks were recorded using a charge-coupled device camera (QuantEM 512SC, Photometrics), and kymographs were produced using MetaMorph software (Molecular Devices). Worms were anesthetized in a drop of M9 containing 10 mM levamisole, transferred to an agarose mount slide, and imaged immediately.

### The bimolecular fluorescence complementation (BiFC) assay

The Venus-based BiFC assay was developed to detect the protein interactions in living worm cilia. For this purpose, we replaced the heat-shock promoter in worm BiFC vectors pCE-BiFC-VN173 and pCE-BiFC-VC155 (Obtained from Addgene, deposited by Dr. Chang-Deng Hu’s lab) with the worm ciliated-cell-specific promoter of arl-13 using *Sph*I and *Xma*I enzyme sites. To visualize BBS-4 and BBS-5 in cilia, worm BBS-4 cDNA was sub-cloned into the VN173 vector, and BBS-5 cDNA into the VC155 vector, respectively. Then, the plasmids were co-injected along with the co-injection marker pRF4 [rol-6 (su1006)] into wild-type worms (15 ng/L for each BiFC plasmid and 100 ng/L pRF4). Fluorescent signals were visualized using a YFP filter.

### GST pull-down

For worm proteins, purified GST or GST-BBS-5 immobilized on Glutathione-Sepharose beads (GE Healthcare) in the binding buffer (20 mM, Tris-HCl, pH 7.4, 150 mM KCl, 5 mM MgCl_2_, 0.5% Triton X-100, and 2 mM β-mercaptoethanol) was incubated with MBP-BBS-4 WT, BBS-4 E107Q, BBS-4 A388E, or BBS-4 fragments (BBS-4 amino-terminus, NT, 1–280 a.a.; BBS-4 carboxyl-terminus, CT, 270 a.a-End) for 4 hours at 4 °C. For human proteins, purified GST or GST-BBS4 was incubated with MBP-BBS5. After five washes with the binding buffer, the samples were subjected to SDS-PAGE and Western blotting analysis with a rat monoclonal anti-Maltose Binding Protein (MBP) antibody (7G4, Sigma).

### Antibodies and reagents

Primary antibodies used in this study include the following: Rabbit polyclonal antibodies against BBS4 (Proteintech), BBS8 (HPA003310, Sigma), Polycystin-2 (H-280, Santa Cruz), Polycystin-2 (Kindly provided by Dr. Feng Qian, University of Maryland); Mouse monoclonal antibody to Acetylated alpha Tubulin (6–11B-1, Sigma); beta-Actin HRP Conjugate (C4, Santa Cruz). All secondary antibodies for immunofluorescence (Highly cross-adsorbed Goat Anti-Rabbit or Anti-Mouse IgG Antibodies conjugated with Alex Fluor 488, 555, or 647) were purchased from Invitrogen.

### Cell Culture, transfection, immunoprecipitation, and immunofluorescence

hTERT RPE-1 (RPE) cells were cultured in DMEM:F12 containing 10% FBS. HeLa and HEK293T cells were maintained in DMEM containing 10% FBS. HEK293T cells were transfected using X-tremeGENE HP DNA Transfection Reagent (Roche).

siRNA duplexes were introduced into cells using Lipofectamine RNAiMAX (Invitrogen). All siRNA oligonucleotides were obtained from Invitrogen. For each gene, two independent siRNA oligonucleotides (O1, O2) were designed. Knockdown Efficiency was routinely verified by Western blotting. All siRNAs except BBS4-O1 gave efficiently knockdowns. Stealth RNAi Negative Control siRNA (Invitrogen) was used as negative control. The sequences of siRNAs are as following:

BBS4-O1: 5′- GGGAGACTTGGACAAGGCCATTGAA -3′;

BBS4-O2: 5′- CCACCTGGATAAGTGTAACCCTTTA -3′;

BBS5-O1: 5′- AAGCTCTCAGCAGAGTGGTGGATAT -3′;

BBS5-O2: 5′- CAGGGACTTTGGGAAGTAATGAGTT -3′;

BBS8-O1: 5′- GGATCAACCTGTGACTGCTTTAAAT -3′;

BBS8-O2: 5′- GAAGAGGCAGCTGATGTCTGGTACA -3′.

Immunoprecipitation was performed as previously described^54^. Briefly, HEK293T cells were co-transfected with Myc-tagged BBS5 and Flag-tagged BBS4 wild type (WT) or A357E mutant. After 24 hours, the cells were lysed in ice-cold IP buffer [20 mM Hepes-KOH, pH7.2, 10 mM KCl, 1.5 mM MgCl2, 1 mM EDTA, 1 mM EGTA, 150 mM NaCl, 0.5% NP-40, Complete Protease Inhibitor Cocktail (Roche), PhosSTOP Phosphatase Inhibitor Cocktail (Roche)]. The cell lysate was then immunoprecipited with normal mouse IgG (Santa Cruz) or anti-Flag antibody (M2, Sigma) followed by Western blotting analysis with anti-Flag and anti-Myc tag (9E10, Santa Cruz) antibodies, respectively.

For indirect immunofluorescence, cells were grown on glass coverslips, fixed with 4% Paraformaldehyde (Electron Microscopy Sciences) for 15 min, permeablized with PBST (Phosphate-buffered saline, with 0.1% Triton X-100), blocked with PBS containing 3% Bovine serum albumin, and stained with appropriate antibodies. To visualize Polycystin-2 on cilia, a published enhanced Immunofluorescence protocol was used^12^. Briefly, cells were prefixed in PBS containing 0.4% paraformaldehyde at 37 °C for 5 min, and extracted with PHEM- Triton buffer (50 mM PIPES, 50 mM HEPES, 10 mM EGTA and 10 mM MgCl_2_, 0.5% Triton X-100, pH 6.9) for 2 min. Then fixed with 4% paraformaldehyde at 37 °C for 5 min, and processed following the regular immunofluorescence protocol. Fluorescence images were acquired using Photoshop software (Adobe).

### Statistical Analyses

Significance was calculated by Student’s t-test using Excel software (Microsoft). P < 0.05, P < 0.01, and P < 0.001 were considered as statistically significant differences. Unless specified, data represent means ± standard deviations (SD) or standard error of the mean (SEM) from at least three independent experiments.

## Additional Information

**How to cite this article**: Xu, Q. *et al.* BBS4 and BBS5 show functional redundancy in the BBSome to regulate the degradative sorting of ciliary sensory receptors. *Sci. Rep.* **5**, 11855; doi: 10.1038/srep11855 (2015).

## Supplementary Material

Supplementary Information
