# Supplementary material for: BBS4 and BBS5 show functional redundancy in the BBSome to regulate the degradative sorting of ciliary sensory receptors
Source: Sci Rep. 2015 Jul 7;5:11855. doi: 10.1038/srep11855 (PMC4493597; doi:10.1038/srep11855)
Supplement: Supplementary Information [file srep11855-s1.pdf]

Supplemental Information

BBS4 and BBS5 show functional redundancy in regulating degradative sorting of ciliary sensory receptors

Qingwen Xu, Yuxia Zhang, Qing Wei, Yan Huang, Yan Li, Kun Ling, Jinghua Hu

Supplemental Figure Legends

Figure S1. BBS-4 and BBS-5 share no conserved domain or structure. (a) BBS-4 and BBS-5 do not share functional domains. The functional domains in worm BBS-4 and BBS-5 and their human orthologs were illustrated by CDD, an online Conserved Domain Database. Hs, *Homo sapiens*; Ce, *Caenorhabditis elegans*. **(b)** BBS-4 and BBS-5 show low similarity in protein sequences. The protein sequences of BBS-4 and BBS-5 and their human orthologs were analyzed by MULTALIN alignment.

Figure S2. IFT-A and IFT-B subcomplexes dissociate in *bbs-4; bbs-5* double mutants as in *bbs-7* mutants. (a) Kymograph analyses showed that IFT-A component CHE-11 moves at slower ~ 0.5 μm/sec in the middle segments of the *bbs-4; bbs-5* or *bbs-7* cilia. **(b)** Kymograph analyses showed that IFT-B component OSM-6 moves at faster ~1.2 μm/sec in the middle segments of the *bbs-4;bbs-5* or *bbs-7* cilia. **(c)** The velocity of IFT-B component OSM-6 remains unchanged in distal segments. Results represented as mean ± SD. n>200. ***p<0.001. N.S., not statistically significant.

Figure S3. The diffusion barrier for membrane proteins is normal in *bbs-4, bbs-5*. (a, b) Membrane protein PPK-1 and ANOctamin (calcium-activated chloride channel) Homolog Y57G11C.37 are normally restricted outside the cilia in WT and *bbs-4; bbs-5* mutants**.** Arrows and arrowheads indicate the base and tip of cilia, respectively. Scale bars, 5 µm.

Figure S4. *bbs-4, bbs-5* mutants does not show non-specific accumulation of overexpressed proteins. (a) The expression level of PDE-1 decreases in *bbs-4, bbs-5* mutants. **(b)** Relative fluorescence intensity of PDE-1-GFP shown indicated as a yellow dashed line in **(a)** was plotted. **(c, d)** The expression level of MKS-5 is unchanged in *bbs-4, bbs-5* mutants. **(d)** Relative fluorescence intensity of MKS-5-mCHERRY shown indicated as a yellow dashed line in **(c)** was plotted. N.S., not statistically significant. Scale bars, 5 µm.

Figure S5. Verification of the expression of VN-BBS-4^{A388E} and BBS-4^{A388E}-GFP.

(a) Non-specific BiFC signal was observed in amphid cell bodies (green arrow head), suggesting BiFC-tagged protein pair was successfully expressed. **(b)** BBS-4^{A388E}-GFP is expressed along the dendrites.

Figure S6. Verification of siRNA-mediated gene knockdowns by immunoblotting. siRNAs were designed to specifically targeting human BBS genes, and their knockdown efficiencies were validated by immunoblotting. **(a, c)** HeLa cells were transfected with either control or indicated siRNAs for 24 h, then transfected with Myc-tagged BBS4 **(a)** or Myc-tagged BBS5 **(c)**

for additional 24 h. O1 and O2 are two independent siRNAs targeting each indicated BBS gene. **(b, d)** RPE cells were transfected with either control or indicated siRNAs for 48 h and further incubated in a serum-free medium for 24 h. Cell lysates were analyzed for immunoblotting using indicated antibodies. Actin was used as a loading control. Arrowheads denote endogenous target proteins. Stars points to non-specific bands.

Figure S1

a

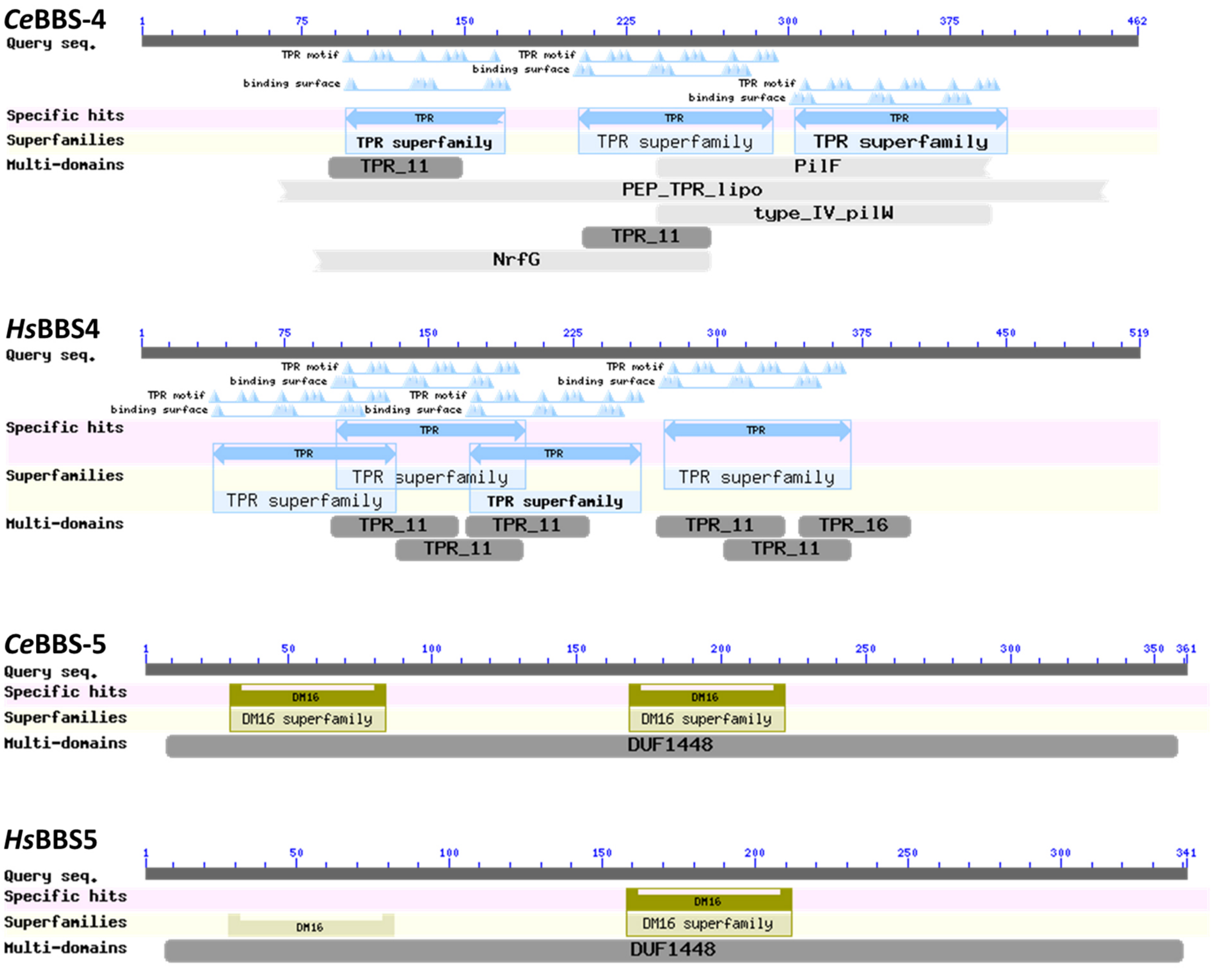

b

```
           1                                                                                                                                       130
  CeBBS-4  measnqdeii gtdvipneqd npeevvpept sldvpppppe rapsapkrve ildcnslngl myhyfaqgdy iecksiigei qskylernea afhvrgliar negeleeame cfhkayelsg knkryfyEtg
   HsBBS4                         maeervatrt qf--pvstes qkprqkkape fpilekqnwl ihlhyirkdy eackavikeq lqetqglcey aiyvqalifr legniqesle lfqtcavlsp qsadnlkQva
  CeBBS-5                                                                                                                                        mErv
   HsBBS5                                                                                                                                          ms
Consensus  .......... .......... .......... .......... .......... .......... .......... .......... .......... .......... .......... .......... .......e..

           131                                                                                                                                     260
  CeBBS-4  rcnfLlgrhq IavEqltKas evmkdNpkVw ywlara-iyh fpaEkvqgkt fNpvesarti lmkpdiAkda tlicflGrlC eelgdTsgAi aaykssIklQ pDnteVMnll gliylRtgqV qegfVqlgnc
   HsBBS4  rslfLlgkhk aaiEVyneaa KLnqkDweIs hnlgVcyiyl kQfNkaqdql hNalnLnr-- -------hdl tyi-mlGkih lLegdldkAi evykkavefs pEntelLTtl gllylqlgiy qkafehlgna
  CeBBS-5  ngediWqDRe IRFDVdhKll rLinGEiqVa kiehVEDTKG NNGDRGtmrV TNLRliWHa- ------Asmp RiNitiGwNa Itgvqskqtt SlvtrnRGis nEAiYVLaKv saTttkFEFI FTttnPaShs
   HsBBS5  vldaLWeDRd VRFDlsaqqm KtrpGEvlId cldsIEDTKG NNGDRGrllV TNLRiLWHs- ------lals RvNvsvGyNC ILnitTrtAn Skl---RG-Q tEAlYILTKc nsT--RFEFI FTnlVPgS-p
Consensus  ....lw.dr. irf#v..k.. kl..g#..!. ....vedtkg nng#rg...v tNlr.lwh.. ......a... r.n...G.nc il...t..a. s.....rg.q .#a.yv$tk. ..t..rfefi ft..vp.s..

           261                                                                                                                                     390
  CeBBS-4  laYdpanSqa iltigSiMqn hsdhdvALnk yrvaadvsdy NgclwnniGI glLarnkpaa shsalkkaaf InplNykISY NlgVlhdimn lhcsalhyik LcteLypqna kavgaMavil shmNddkNar
   HsBBS4  ltYdptnyka ilaagSmMqt hgdfdvALtk yrvvacavPE spplwnniGm cffgkkkyva aisclkrany lapfDwkIlY NlglvhLTmq qyasafhfls aAinfQpkmg elymlLaval tnlEdiENak
  CeBBS-5  kLFntisSis RAYETtKMYR elKmRgvfIr edgtLkiLPQ EtiieKvNGV WNLStetGsL GvFvITNiRv VWyAEMNIgY NVSVPYiTly SvrvReSKFG MALVLEttts SGeYVLGFrv DPaErLQNll
   HsBBS5  rLFtsvmavh RAYETSKMYR dfKlRsALI- qnkqLrlLPQ EhvydKiNGV WNLSsdqGnL GtFfITNvRi VWhANMNdSF NVSIPYLqir SikiRdSKFG LALViEssqq SGgYVLGFki DPvEkLQEsv
Consensus  .l%....s.. rayetskMyr ..k.r.ali. ....l..lpq e....k.nGv wnls...g.l g.f.itn.r. vw.a#mnis% Nvsvpylt.. s...r.skfg lalvle.... sg.yv$gf.. dp.#.lq#..

           391                                                                                                                                     520
  CeBBS-4  laykksielk k-nPstilnY aifeyrmkDv ta---asnal klykDlqasg vKcsaNnket anllEsvlkQ pdQsaeq
   HsBBS4  rayaeavhld kcnPlvnlnY avllyNqgE- -----kknal aQyQEmekkv SllkdNssle FdsemvemaQ klgaalqvgE alvwtkPvKD pkskHqttst skpasfqqpl gsnqalgqam ssaaayrtlp
  CeBBS-5  KtvqSLHKah llkPIFGVsY vkEiaEKvEP rkdddEidii dNnEDDVEID dKirpDAFas YFdgEhtatD ekQlPVlnpE LGLAIEPirN GFTLHdLWni hvdv
   HsBBS5  KeinSLHKvy sasPIFGVdY --EmeEKpQP l----Ealtv eQiQDDVEID SdghtDAFva YFadgnkqqD re--PVfseE LGLAIEklKD GFTLqgLWev ms
Consensus  k...slhk.. ...Pifgv.Y ..e..ek.#p .....e.... .q.q#dveid sk...#af.. yf..e....# ..q.pv...e lglaiep.kd gftlh.lw.. .......... .......... ..........

           521                             557
  CeBBS-4
   HsBBS4  sgaggtsqft kppslplepe pavessptet seqirek
  CeBBS-5
   HsBBS5
Consensus  .......... .......... .......... .......
```

Figure S2

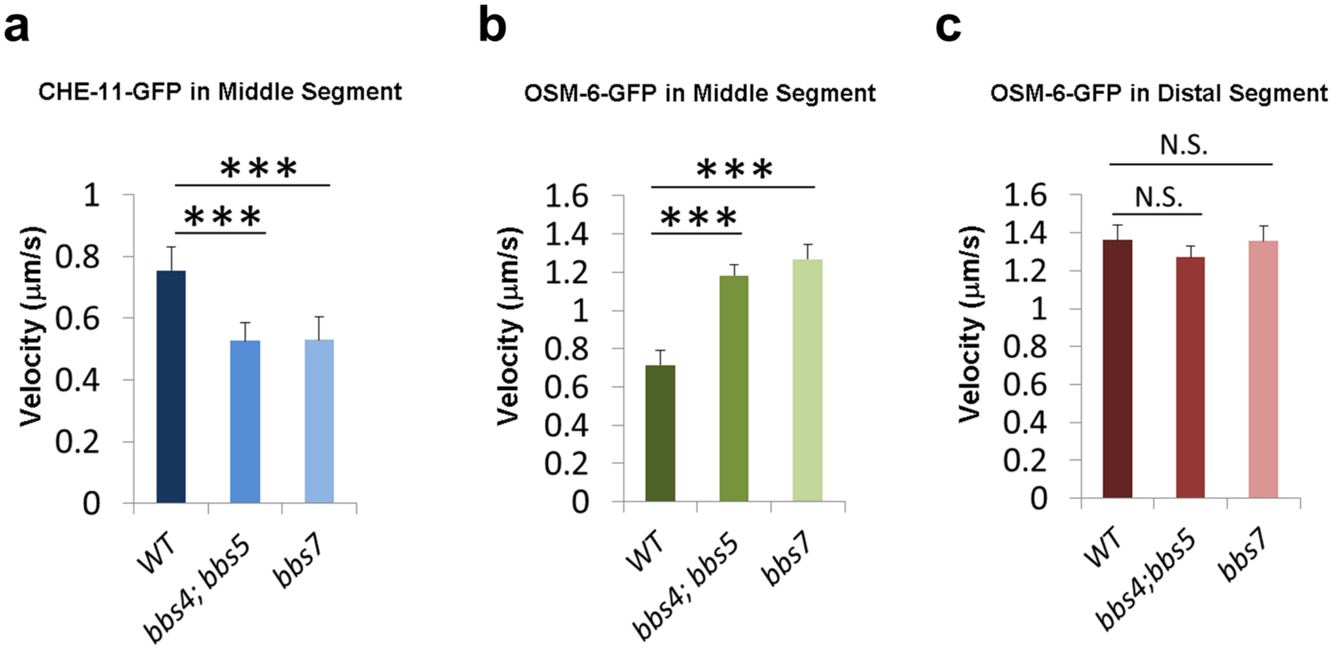
a
CHE-11-GFP in Middle Segment
Velocity (μm/s)
1
0.8
0.6
0.4
0.2
0
***
***
WT
bbs4; bbs5
bbs7
b
OSM-6-GFP in Middle Segment
Velocity (μm/s)
1.6
1.4
1.2
1
0.8
0.6
0.4
0.2
0
***
***
WT
bbs4; bbs5
bbs7
c
OSM-6-GFP in Distal Segment
Velocity (μm/s)
1.6
1.4
1.2
1
0.8
0.6
0.4
0.2
0
N.S.
N.S.
WT
bbs4;bbs5
bbs7

Figure S3

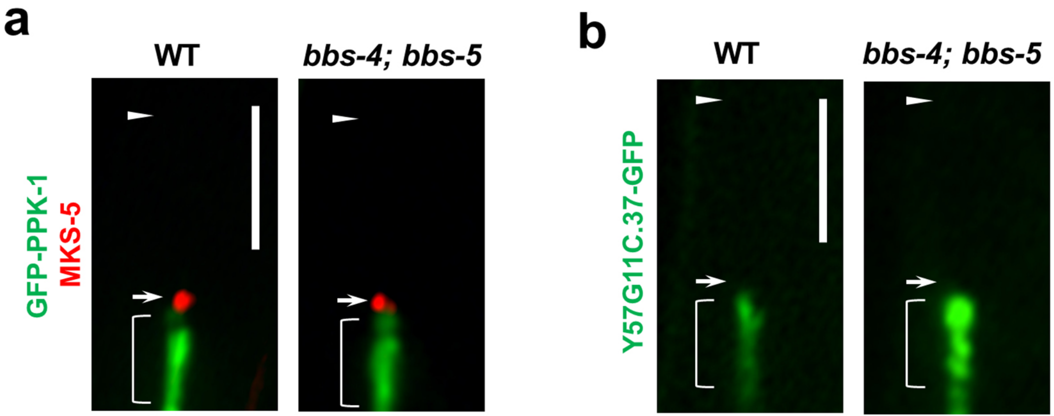

Figure S4

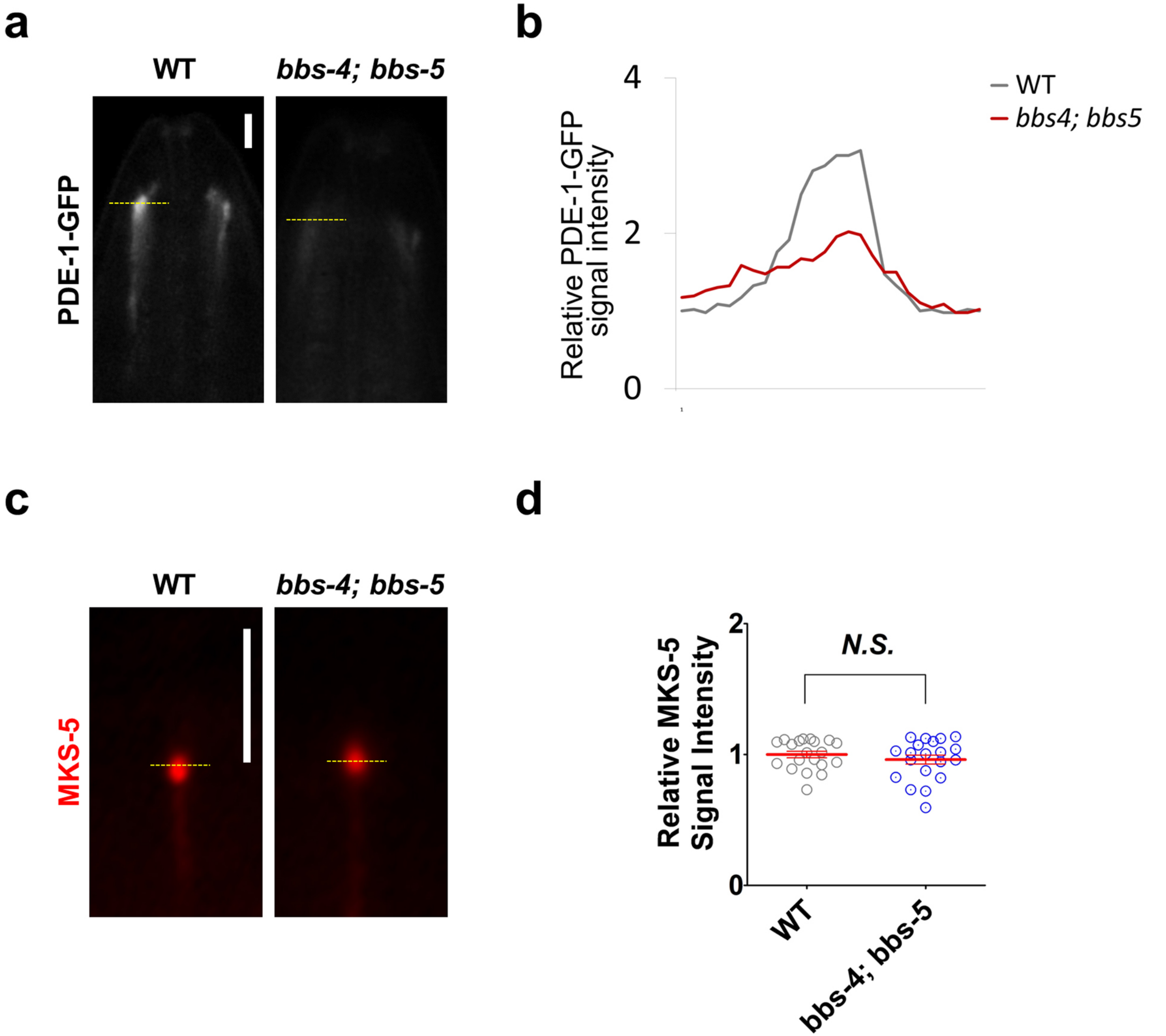

a
WT
bbs-4; bbs-5
PDE-1-GFP
b
Relative PDE-1-GFP signal intensity
4
2
0
WT
bbs4; bbs5
c
WT
bbs-4; bbs-5
MKS-5
d
Relative MKS-5 Signal Intensity
2
1
0
N.S.
WT
bbs-4; bbs-5

Figure S5

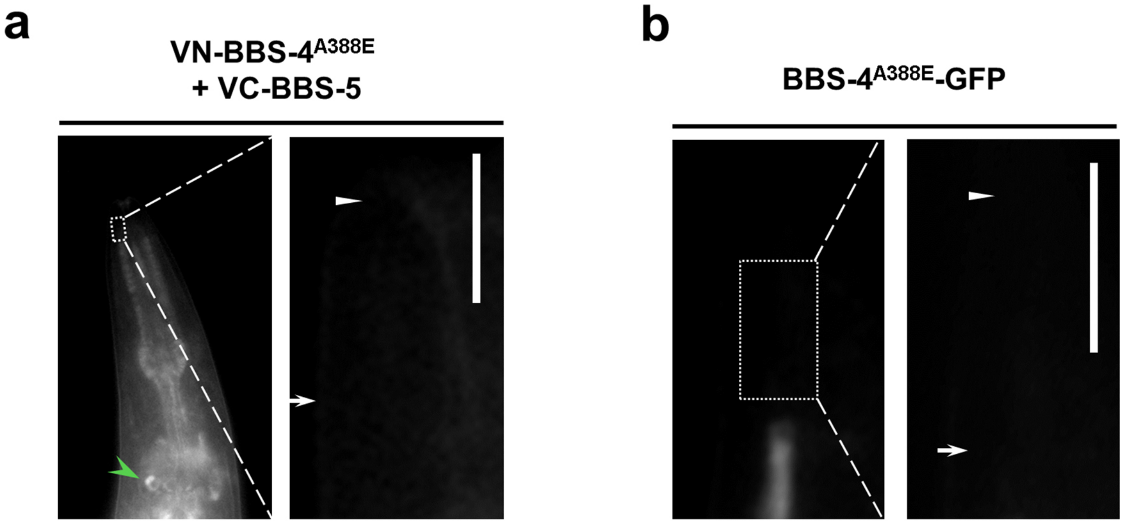

Figure S6

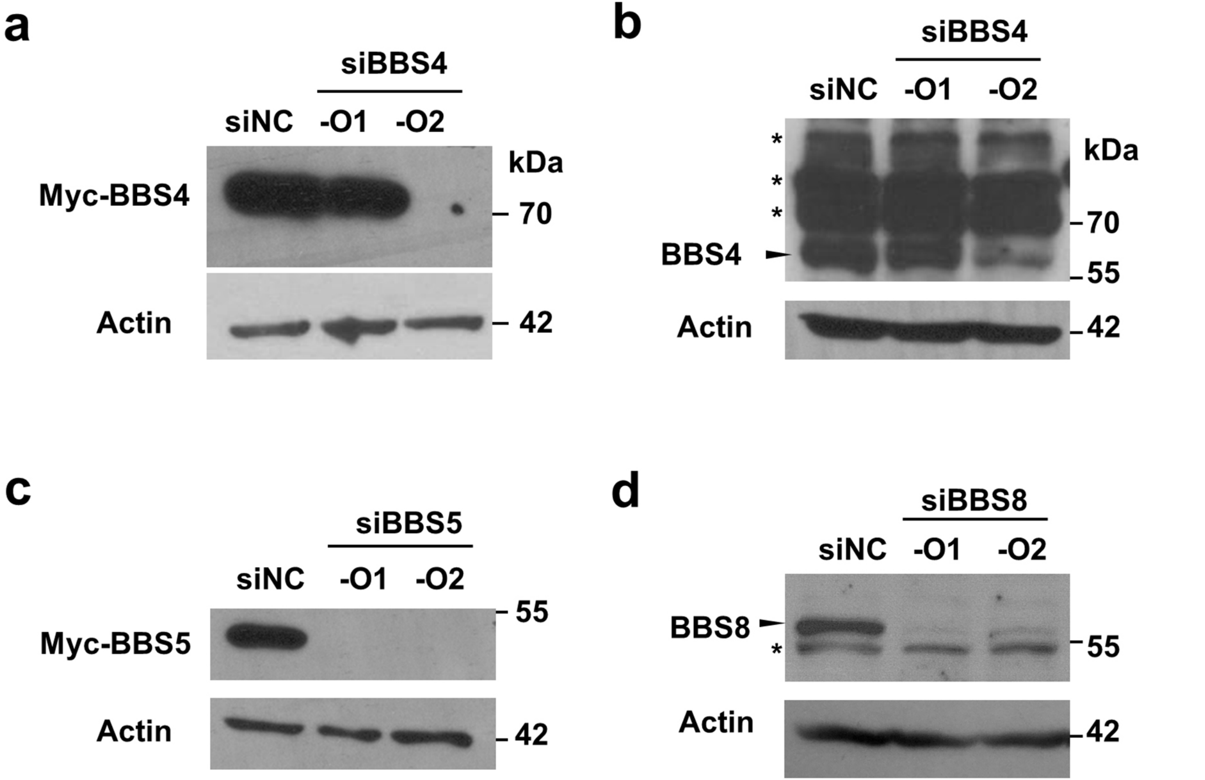

a
siBBS4
siNC
-O1
-O2
kDa
Myc-BBS4
70
Actin
42
b
siBBS4
siNC
-O1
-O2
kDa
*
*
*
70
BBS4
55
Actin
42
c
siBBS5
siNC
-O1
-O2
55
Myc-BBS5
Actin
42
d
siBBS8
siNC
-O1
-O2
BBS8
*
55
Actin
42
